# Economic Abuse and Care-seeking Practices for HIV and Financial Support Services in Women Employed by Sex Work: A Cross-Sectional Baseline Assessment of a Clinical Trial Cohort in Uganda

**DOI:** 10.1177/08862605221093680

**Published:** 2022-05-05

**Authors:** Larissa Jennings Mayo-Wilson, Bing-Jie Yen, Proscovia Nabunya, Ozge Sensoy Bahar, Brittanni N. Wright, Joshua Kiyingi, Prema L. Filippone, Abel Mwebembezi, Joseph Kagaayi, Yesim Tozan, Josephine Nabayinda, Susan S. Witte, Fred M. Ssewamala

**Affiliations:** 1 41474 University of North Carolina Gillings School of Global Public Health, Chapel Hill, NC, USA; 2 Community, Global and Public Health Division, 15851 Johns Hopkins University School of Nursing, Baltimore, MD, USA; 3 Department of Applied Health Science, Center for Sexual Health Promotion, 41473 Indiana University School of Public Health, Bloomington, IN, USA; 4 51503 Washington University in St. Louis, Brown School, St. Louis, MO, USA; 5 International Center for Child Health and Development, Masaka, Uganda; 6 139058 Columbia School of Social Work, New York, NY, USA; 7 Reach the Youth Uganda, Kampala, Uganda; 8 561068 Rakai Health Sciences Program, Kalisizo, Uganda; 9 550517 New York University College of Global Public Health, New York, NY, USA

**Keywords:** HIV, sexual risk behaviors, economic abuse, female sex workers, violence, Uganda

## Abstract

Economic hardship is a driver of entry into sex work, which is associated with high HIV risk. Yet, little is known about economic abuse in women employed by sex work (WESW) and its relationship to uptake of HIV prevention and financial support services. This study used cross-sectional baseline data from a multisite, longitudinal clinical trial that tests the efficacy of adding economic empowerment to traditional HIV risk reduction education on HIV incidence in 542 WESW. Mixed effects logistic and linear regressions were used to examine associations in reported economic abuse by demographic characteristics, sexual behaviors, HIV care-seeking, and financial care-seeking. Mean age was 31.4 years. Most WESW were unmarried (74%) and had less than primary school education (64%). 48% had savings, and 72% had debt. 93% reported at least one economic abuse incident. Common incidents included being forced to ask for money (80%), having financial information kept from them (61%), and being forced to disclose how money was spent (56%). WESW also reported partners/relatives spending money needed for bills (45%), not paying bills (38%), threatening them to quit their job(s) (38%), and using physical violence when earning income (24%). Married/partnered WESW (OR = 2.68, 95% CI:1.60–4.48), those with debt (OR = 1.70, 95% CI:1.04–2.77), and those with sex-work bosses (OR = 1.90, 95% CI:1.07–3.38) had higher economic abuse. Condomless sex (*β* = +4.43, *p* < .05) was higher among WESW experiencing economic abuse, who also had lower odds of initiating PrEP (OR = .39, 95% CI:.17–.89). WESW experiencing economic abuse were also more likely to ask for cash among relatives (OR = 2.36, 95% CI:1.13–4.94) or banks (OR = 2.12, 95% CI:1.11–4.03). The high prevalence of HIV and economic abuse in WESW underscores the importance of integrating financial empowerment in HIV risk reduction interventions for WESW, including education about economic abuse and strategies to address it. Programs focusing on violence against women should also consider economic barriers to accessing HIV prevention services.

## Introduction

Women employed by sex work (WESW) are disproportionately affected by human immunodeficiency viruses (HIV) (UNAIDS, 2019). According to the Joint United Nations Programme on HIV and AIDS (UNAIDS), the risk of acquiring HIV for people who are employed by sex work is 21 times higher than the risk of acquiring HIV in adults, aged 15–49, who do not exchange sex for money or other non-monetary items (UNAIDS, 2019). In sub-Saharan Africa, WESW and their clients account for 39% of all new HIV infections (UNAIDS, 2019). Inconsistent condom use in the absence of anti-retroviral medication accounts for the most proximal risk for HIV acquisition and transmission in WESW (Couture et al., 2020). However, other contributing factors to HIV risk in WESW include the high rate of physical and sexual violence that WESW experience by their sex partners, which impedes uptake of safer sex behaviors (Couture et al., 2020). Studies have shown that physical and sexual violence against WESW, by sex partners or family members, is associated with barriers to condom access (Goldenberg et al., 2020; Logie et al., 2020) and lower condom use (Decker et al., 2020; Goldenberg et al., 2020; Logie et al., 2020; Peitzmeier et al., 2020) due to women’s limited ability to enforce safer sex. Yet, while efforts to address physical and sexual violence against women have increased substantially over the years (Marshall et al., 2018), there remains scarce empirical evidence on the correlates and consequences of economic abuse as another form of violence perpetrated against women (Adams et al., 2015; Antai et al., 2014; Christy et al., 2020; Tenkorang & Owusu, 2019).

Economic abuse is defined as the financial control and exploitation of a person’s ability to acquire, use, and maintain economic resources (i.e., monetary assets, education, employment) (Adams et al., 2015; Anitha, 2019; Sanders, 2015; Voth Schrag & Ravi, 2020). Economic abuse undermines a person’s economic security and potential for economic self-sufficiency (Adams et al., 2015; Anitha, 2019; Sanders, 2015; Voth Schrag & Ravi, 2020). Women experiencing economic abuse have been found to have significant economic hardship as evidenced by decreased economic self-sufficiency (Postmus et al., 2011, 2012), limited access to credit (Littwin, 2012), increased financial strain (Adams et al., 2008; Kutin et al., 2017), increased food insecurity (Power, 2006), and lost productivity (Moe & Bell, 2004; Swanberg et al., 2005). Economic abuse is increasingly viewed as a form of violence against women in the context of women’s paid and unpaid labor (Anitha, 2019; Christy et al., 2020; Postmus et al., 2020). When enacted, economic abuse includes tactics of economic control used by sex partners or family members, such as withholding earnings, restricting or interfering with employment, regulating access to money, denying access to financial information, limiting purchasing decisions, or blaming a woman for spending money on personal or family needs (Adams et al., 2015; Davila et al., 2017; Stylianou, 2018; Tenkorang & Owusu, 2019; Usta et al., 2013). Economic abuse also includes financially exploitive behaviors, such as stealing money, refusing to work (or earn income), and generating debt in another person’s name (Adams et al., 2015; Davila et al., 2017; Tenkorang & Owusu, 2019).

Economic hardship is a key driver of entry into sex work (George et al., 2019; Gertler et al., 2005; Rao et al., 2003; Sagtani et al., 2014). Yet, little is known about experiences of economic abuse in WESW. The available studies on economic abuse have primarily focused on financial mistreatment of elderly populations in high-income countries (Acierno et al., 2019; Antai et al., 2014). Questions remain about the extent to which WESW in low-income countries experience economic abuse, and whether certain WESW are at greater risk of being economically abused compared to other WESW (Antai et al., 2014; Yau et al., 2020). In addition, given the disproportionately high HIV incidence in WESW, there is growing interest on whether experiencing economic abuse exacerbates women’s vulnerability to HIV. Prior studies among WESW have examined the association of economic insecurity measures, such as debt, income, or housing, on unprotected sex (Barreto et al., 2017; Fehrenbacher et al., 2016; Lim et al., 2019; Reed et al., 2010) and incidence of sexually transmitted infections (Reed et al., 2010). Yet, the relationship between economic abuse and uptake of HIV prevention practices, such as condom use or care-seeking for HIV testing and medications (i.e., ARV or PrEP), is not well known (George et al., 2019). WESW who experience economic abuse may face financial barriers in seeking HIV services or experience increased violence by partners or family members who disapprove of real and/or perceived costs of seeking HIV prevention and care services (i.e., condom purchases, travel costs to clinics, medication-related expenses). WESW who experience economic abuse may also face difficulties in seeking WESW-focused financial assistance programs.

This study comprised of three main objectives. The first objective was to determine the prevalence of economic abuse among WESW who were recently enrolled in a randomized clinical trial testing a combination intervention that included HIV risk reduction education and economic empowerment components in southern Uganda. The second objective was to identify demographic characteristics associated with reported economic abuse in WESW. The third objective was to examine the association of economic abuse with engagement in sexual risk behaviors, care-seeking practices for HIV, and care-seeking practices for financial support. Results are used to discuss programming implications for improving economic and sexual health in WESW.

## Methods

### Study Design

This study used baseline data from the Kyaterekera Project, a multisite, longitudinal randomized clinical trial that tests the efficacy of adding savings, financial literacy, and mentorship to traditional HIV risk reduction (HIVRR) education on reducing new incidence of HIV and other sexually transmitted infections (STIs) among a cohort of 990 WESW in the greater Masaka region in southern Uganda, in partnership with the Rakai Health Sciences Program and Reach the Youth. A detailed description of the study’s methodology and ethics approval is described in a previously published protocol manuscript (Ssewamala et al., 2019). In sum, WESW were eligible to participate if they were: aged 18  years or older; reported having engaged in vaginal or anal intercourse in the past 30  days in exchange for money, alcohol, or other goods; and reported having had at least one episode of unprotected sexual intercourse in the past 30 days with either a paying, non-paying, casual, or regular sexual partner (Ssewamala et al., 2019). We recruited self-identified WESW from October 2019 to February 2020, at which time recruitment activities were suspended due to the COVID-19 pandemic. WESW recruited into the study up to that time were interviewed at the time of study enrollment (baseline) and retained for follow-up assessments in 6-month intervals up to 24 months, resulting in an enrolled sample of 542 WESW rather than the target sample of 990 WESW. WESW were recruited using community and peer liaisons from the International Center for Child Health and Development (ICHAD). All WESW were administered informed consent prior to study enrollment.

### Setting

The national HIV prevalence in Uganda among adults, aged 15–49, is 5.8% with the greater Masaka region having notably higher HIV prevalence of 12.0% (UNAIDS, 2014; Uganda AIDS Commission Ministry of Health UNAIDS, 2013). HIV prevalence is over 8 times higher among Ugandan WESW (61%) as compared to Ugandan adults who are not employed by sex work (Ssembatya et al., 2015; Vandepitte et al., 2011), and 77% of WESW in the region reported a new STI diagnosis in the prior 12 months (Matovu & Ssebadduka, 2012). There are an estimated 1895 registered WESW (i.e., recognized as sex workers by local jurisdiction) within targeted “hotspots” or high HIV prevalence areas of the study region.

### Outcome Measure

The primary outcome of this study was economic abuse. To measure economic abuse, enrolled women were asked 12 questions about whether a current or past sex partner had ever hurt them financially since the start of their relationship based on the following items: (i) made you ask them for money; (ii) demanded to know how money was spent; (iii) demanded that you give them receipts and/or change when money was spent; (iv) kept financial information from you; (v) made important financial decisions without talking with you first; (vi) threatened to make you leave job/work; (vii) demanded that you quit your job/work; (viii) beat you up if you said you needed to go to work; (ix) did things to keep you from going to your job/work; (x) spent money you needed for rent and other bills; (xi) paid bills late or not pay bills that were in your name or both of your names; and (xii) borrowed money or purchased things on credit under your name. These items were informed by and used in prior studies of economic abuse and validated in adult populations, including women, in the U.S. and other African, Asian, and South American countries (Adams et al., 2015; Anitha, 2019; Christy et al., 2020; Davila et al., 2017; Postmus et al., 2011, 2012, 2020; Stylianou, 2018; Tenkorang & Owusu, 2019; Voth Schrag & Ravi, 2020; Yau et al., 2020). For each of the 12 items, women were asked to select one of five response options of “never,” “hardly ever,” “sometimes,” “often,” and “quite often.” Current or past sex partners were defined as any individual in the WESW’s lifetime with whom she had engaged in vaginal, anal, or oral sexual intercourse, regardless of whether for pay or not for pay (i.e., spouse, lover, romantic partner, boyfriend, girlfriend, sex work clients). WESW were coded as having ever experienced the economic abuse item (yes = 1) if they stated sometimes, often, or quite often. WESW were coded as having not experienced the economic abuse item (no = 0) if they stated hardly ever or never. Following their reports of economic abuse by current or past sex partners, WESW were asked the same 12 questions regarding economic abuse by a non-sex partner family member (i.e., parent, sibling, etc.).

Secondary outcomes included sexual risk behaviors, care-seeking practices for HIV, and care-seeking practices for financial support. Sexual risk behaviors were measured using two variables: condomless sex and sex under the influence of alcohol or drugs. WESW were asked how many times they had vaginal or anal sex in the last 90 days with their two most recent sex partners during that time period and the number of times a male or female condom was used. WESW were coded as having condomless sex (yes = 1) if they reported condom use <100% of all sex acts during the specified period. WESW who reported an equal number of condom use times as number of sex acts (i.e., 100%) were coded as not having condomless sex (no = 0). WESW were also asked how many times they and/or their most recent sexual partner had drunk alcohol or taken drugs before having sex. WESW who reported that they and/or their two most recent sexual partners had sex under the influence of alcohol/drugs one or more times in the last 90 days were coded as yes (=1). WESW who reported zero times for both themselves and their most recent sex partner was coded as no (=0).

Care-seeking practices for HIV were measured using three variables: receipt of HIV test services, initiation of anti-retroviral treatment (ART) in HIV-positive women, and initiation of pre-exposure prophylaxis (PrEP) in HIV-negative women. WESW were asked if they had ever been tested for HIV, and, if so, during what month and year. WESW who reported receiving an HIV test within 90 days of the date of interview were coded as 1 (=yes). WESW who had never received an HIV test or who had received an HIV test >90 days of the date of interview were coded as 0 (=no). Among WESW who had ever tested for HIV, we asked if they had ever received a positive HIV test result, and, if so, had they initiated antiretroviral therapy (ART) (yes = 1 or no = 0). ART initiation was not measured in HIV-uninfected WESW. For WESW who had never received a positive HIV test result, we asked if they had received a prescription for HIV pre-exposure prophylaxis (PrEP) or a daily pill to help prevent HIV (yes = 1 or no = 0). PrEP initiation was not measured in HIV-infected WESW. Care-seeking practices for financial support were measured using two variables: receipt of money from relatives and friends and receipt of credit/debt from a lending institution (yes = 1 or no = 0) in the last 90 days. Information was also obtained on each WESW demographic characteristics, including women’s age, marital status, highest level of education, adult household size, monthly income, having savings, having a sex work boss/manager, and having non-sex work employment.

### Analysis

Data were analyzed using STATA SE, Version 15 (Stata Corporation, College Station, TX). The analytical sample included all WESW who were enrolled in the study up until the end of recruitment. We first examined the distribution of demographic characteristics and the distribution of economic abuse items by sex partners and by family members. WESW could report abuse by both types of abusers yielding non-mutually exclusive groups. Therefore, one-sample binomial tests were used to examine whether the proportion of reported economic abuse by family members significantly differed from a hypothesized value equivalent to the proportion of reported economic abuse by sex partners for each item.

To examine total prevalence of economic abuse, we calculated the sum of reported economic abuse items, ranging from a score of 0 to 24, for each WESW. WESW were categorized as having experienced economic abuse if they reported ≥1 items. However, given the overwhelming majority of WESW who experienced one or more economic abuse items (93%), WESW were also ranked according to the total economic abuse score and divide into tertiles (i.e., high, medium, and low) using the *xtile* function in STATA. We chose to analyze the data using tertiles to enhance the interpretation of findings in assessing WESW with varying levels of exposure to economic abuse. However, we also calculated the mean number of reported economic abuse items and standard deviation (i.e., continuous measure) to assess overall distribution of economic abuse among WESW. Data were hierarchically structured as WESW were nested within sites (*n* = 19), requiring a multilevel approach. Bivariate and multivariable mixed effects logistic and linear regressions were used to examine differences in the association of reported economic abuse by demographic characteristics, sexual behaviors, HIV care-seeking, and financial care-seeking. To do so, we estimated the crude odds ratio (OR) and adjusted odds ratio (aOR) of experiencing one or more episodes of economic abuse versus no report of economic abuse for each demographic, sexual, and care-seeking variable, adjusting for age, education, and partnership status in multivariable models. We similarly examined the crude and adjusted OR of high and medium levels of economic abuse, respectively, versus the reference group of low economic abuse. The small number of WESW who reported no economic abuse were included in the lowest tertile. Linear regressions were used to examine differences in the mean number of reported condomless sex acts in the past 3 months and the mean number of reported sex acts under the influence of drugs/alcohol in the past 3 months among WESW experiencing high economic abuse compared to those experiencing low economic abuse. All analyses were considered statistically significant at *p* < .05 or when the 95% confidence interval (CI) did not include the null odds ratio of 1.0.

## Results

### Sample Demographic Characteristics

Table 1 describes the sample’s demographic characteristics. A total of 542 WESW were enrolled in the study (Table 1). The mean age was 31.4 years (±7.2), ranging from 18 to 55 years. The majority of WESW were single/unmarried (74%, *n* = 403), had less than primary school education (64%, *n* = 344), and lived in a one-adult household (59%, *n* = 317). Financial status varied within the sample. Less than half of WESW reported having savings (48%, *n* = 260), but 72% (*n* = 388) reported having debt. All women who were employed by sex work had an average of 31.3 paying clients (+47.1) in the last 30 days and average individual monthly income of $60.30 US dollars (equal to approximately 213,820 Ugandan shillings, where 1 USD = 3546.000 UGX, 2021). Having a sex work boss/manager was uncommon (16%, *n* = 88), and few WESW reported other non-sex work employment in the last 12 months (2%, *n* = 8).

**Table 1.** Demographic Characteristics of Women Employed by Sex Work at Time of Study Enrollment (*N* = 542).

Demographic Characteristic	*N*	(n/*N*) %
Total sample	542	100%
Mean age in years (±SD)	31.4 (±7.2)	––
Age range in years	18–55	––
Age in years		
18–29	232	42.8%
≥30	310	57.2%
Partnership status		
Single/separated^a^	403	74.4%
Married/partnered^b^	139	25.7%
Highest level of education		
Less than primary education	344	63.5%
Primary school or more	198	36.5%
Adult household size^c^		
One adult	317	58.5%
Two or more adults	225	41.5%
Earns more than sample mean monthly income in USD^d^		
Yes	176	32.5%
No	366	67.5%
Has savings		
Yes	260	48.0%
No	282	52.0%
Has debt		
Yes	388	71.6%
No	154	28.4%
Has a sex work boss/manager		
Yes	88	16.2%
No	454	83.8%
Mean number of paying clients in the past 30 days (±SD)	31.3 (±47.1)	––
Has other non-sex work employment in last 12 months		
Yes	8	1.5%
No	534	98.5%

^a^Not partnered includes separated, divorced, widowed, and single/never married.

^b^Partnered includes legal marriage, common law marriage, and committed non-marital relationship.

^c^Ranges from 1 to 18 and includes enrolled WESW as one adult.

^d^Mean reported individual monthly income is $60.30 USD.

### Prevalence of Economic Abuse

Table 2 describes the prevalence of reported economic abuse items by WESW’s sex partners and/or family members. The prevalence of economic abuse was high. Ninety-three percent (93%, *n* = 503) reported at least one economic abuse incident with an average of 7.3 (±5.7) reported items (Table 2). The four most commonly reported economic abuse items by the majority of WESW included being forced to ask for money (80%, *n* = 435), having financial information kept from them (61%, *n* = 329), having important financial decisions made without talking to them first (58%, *n* = 316), and demanding to disclose how their money was spent (56%, *n* = 301). Over a third of WESW also reported their sex partner and/or family member spent money they needed for rent or other bills (45%, *n* = 245), demanded receipts for money spent (39%, *n* = 213), did not pay bills that were in both of their names (38%, *n* = 208), and demanded or threatened them to quit their job/work, respectively (38%, *n* = 203; 37%, *n* = 198). More serious forms of economic abuse were prevalent in approximately one quarter of WESW, including physical violence for women who attempt to earn income (24%, *n* = 132) and financial exploitation in the form of borrowing money/credit in women’s name (26%, *n* = 141). WESW were significantly more likely to report economic abuse by their sex partner as compared to their family members for 11 of the 12 economic abuse items (*p* < .05). The Cronbach’s alpha internal reliability measure of the economic abuse items was .8497.

**Table 2.** Prevalence of Reported Economic Abuse Items by Sex Partner and/or by Family Member Among Women Employed by Sex Work at Time of Study Enrollment (*N* = 542).

Economic Abuse Item	Total		By Type of Abuser				
*N* = 542	By Sex Partner And/Or Family Member		By Sex Partner Only		By Family Member Only		Test by Abuser type^a^
Item-level measures	n	%	n	%	n	%	*p*-value
Made you ask them for money	435	80.3	392	72.3	229	42.3	0
Demanded to know how money was spent	301	55.5	273	50.4	144	26.6	0
Demanded you give them receipts or change for money spent	213	39.3	185	34.1	98	18.1	0
Kept financial information from you	329	60.7	298	55.0	177	32.7	0
Made financial decisions without talking with you first	316	58.3	276	50.9	166	30.6	0
Threatened to make you leave job/work	198	36.5	175	32.3	85	15.7	0
Demanded that you quit your job/work	203	37.5	179	33.0	94	17.3	0
Beat you up if you said you needed to go to job/work	132	24.4	111	20.5	62	11.4	0
Did things to keep you from going to your job/work	178	32.8	157	29.0	74	13.7	0
Spent money you needed for rent or other bills	245	45.2	218	40.2	101	18.6	0
Paid bills late or did not pay bills in your name or both of your names	208	38.4	188	34.7	88	16.2	0
Borrowed money or purchased things on credit under your name	141	26.0	105	19.4	94	17.3	0.254
Compositive measures							
Range (min, max)	(0, 24)		(0, 12)		(0, 12)		—
Reported ≥1 economic abuse items	92.8%		—		—		—
Mean # economic abuse items reported (±SD)	7.3 (±5.7)		4.7 (±3.4)		2.6 (+3.1)		0
Mean # economic abuse items reported (+SD)							
Lowest tertile	2.0 (±1.4)^b^		1.4 (±1.2)		0.6 (±0.9)		0
Medium tertile	6.5 (±1.2)^c^		4.7 (±1.8)		1.8 (±1.5)		0
Highest tertile	13.9 (±4.4)^d^		8.4 (±2.5)		5.6 (±3.5)		0

^a^Includes one-sample binomial and one-sample *t*-tests for categorial and continuous variables, respectively, against a hypothesized value equivalent to the other abuser type.

^b^Includes lowest third of summed economic abuse scores, ranging from 0 to 4.

^c^Includes middle third of scores, ranging from 5 to 8.

^d^Includes highest third of scores, ranging from 9 to 24.

### Demographic Characteristics Associated with Economic Abuse

Table 3 reports the crude and adjusted odds ratio (OR) of reported economic abuse by WESW’s demographic factors. Given the high prevalence of any economic abuse (93%, *n* = 503), no demographic factors were significantly associated with any experience of abuse. However, certain demographic characteristics were significantly associated with experiencing high and medium levels of economic abuse as compared to low levels of economic abuse (by tertiles). In adjusted analyses, married/partnered WESW (OR = 2.68, 95%CI: 1.60–4.48), those with debt (OR = 1.70, 95%CI: 1.04–2.77), and those with a sex work boss/manager (OR = 1.90, 95%CI:1.07–3.38) had significantly greater odds of experiencing higher economic abuse as compared to single WESW, those without debt, and those without a sex work boss/manager, respectively. Having above average individual monthly income was a protective factor against higher reported economic abuse. WESW with above average monthly income had 50% lower odds of reporting high economic abuse (OR = .50, 95%CI: .31–.80) as compared to women with below average monthly income. In adjusted analyses, WESW’s age (OR = 0.85, 95%CI: .54–1.34), education level (OR = 1.13, 95%CI: 0.71–1.80), household size (OR = 1.26, 95%CI: .82–1.95), and savings (OR = .92, 95%CI: .60–1.41) were not significantly associated with differential reports of economic abuse.

**Table 3.** Crude and Adjusted Odds Ratios (OR) of Demographic Factors Associated with Reported Economic Abuse (EA) in Women Employed by Sex Work by Total and by Level of Exposure (*N* = 542).

	By Any EA			By Level of EA						
	Yes	No	Crude OR (95% CI)	Low	Med	High	Crude OR (95% CI)		Adjusted OR (95% CI)^a^	
	%	%	Any versus None	%	%	%	Med Versus Low	High Versus Low	Med Versus Low	High Versus Low
Total (*N*)	*n* = 503 (92.8%)	*n* = 39 (7.2%)	—	*n* = 189 (34.9%)	*n* = 178 (32.8%)	*n* = 175 (32.3%)	—	—	—	—
Age										
≥30 years	93.6%	6.4%	1.00	34.8%	31.3%	33.9%	1.00	1.00	1.00	1.00
18–29 years	91.8%	8.2%	.77 (.40, 1.49)	34.9%	34.9%	30.2%	1.08 (.71, 1.65)	.87 (.56, 1.33)	1.04 (.66, 1.64)	.85 (.54, 1.34)
Partnered										
No	91.8%	8.2%	1.00	39.0%	29.0%	32.0%	1.00	1.00	1.00	1.00
Yes	95.7%	4.3%	2.04 (.83, 5.05)	23.0%	43.9%	33.1%	2.70 (1.62, 4.51)***	1.74 (1.04, 2.92)*	2.68 (1.60,4.48)***	1.72(1.02, 2.89)*
Education										
< Primary	93.0%	7.0%	1.00	36.9%	30.2%	32.9%	1.00	1.00	1.00	1.00
≥ Primary	92.4%	7.6%	.91 (.46, 1.78)	31.3%	37.4%	31.3%	1.43 (.93, 2.21)	1.10 (.70, 1.71)	1.41 (.88, 2.20)	1.13 (.71, 1.80)
Household size										
1	91.8%	8.2%	1.00	37.2%	32.2%	30.6%	1.00	1.00	1.00	1.00
≥2	94.2%	5.8%	1.43 (.71, 2.87)	31.6%	33.8%	34.7%	1.21 (.79, 1.85)	1.33 (.87, 2.03)	1.05 (.67, 1.66)	1.26 (.82, 1.95)
> Mean income										
No	93.2%	6.8%	1.00	32.5%	30.6%	36.9%	1.00	1.00	1.00	1.00
Yes	92.0%	8.0%	.85 (.43, 1.69)	39.8%	37.5%	22.7%	1.02 (.66, 1.58)	.49 (.31, .79)**	.87 (.55, 1.37)	.50 (.31, .80)**
Non-sex work										
No	92.7%	7.3%	NC^b^	34.6%	33.3%	32.1%	NC^b^	1.00	NC^b^	1.00
Yes	100%	0%		50%	0%	50%		1.11 (.27, 4.63)		1.07 (.26, 4.52)
Has savings										
No	92.9%	7.1%	1.00	35.5%	30.8%	33.7%	1.00	1.00	1.00	1.00
Yes	92.7%	7.3%	.95 (.49, 1.84)	34.2%	35.0%	30.8%	1.16 (.76, 1.76)	.92 (.61, 1.41)	1.06 (.69, 1.64)	.92 (.60, 1.41)
Has debt										
No	91.6%	8.4%	1.00	39.6%	26.6%	33.8%	1.00	1.00	1.00	1.00
Yes	93.2%	6.7%	1.28 (.63, 2.57)	33.0%	35.3%	31.7%	1.60 (1.00, 2.56)*	1.10 (0.70, 1.73)	1.70 (1.04, 2.77)*	1.11 (0.71, 1.74)
Sex work boss										
No	92.3%	7.7%	1.00	36.1%	33.5%	30.4%	1.00	1.00	1.00	1.00
Yes	95.5%	4.5%	1.74 (.60, 5.07)	28.4%	29.5%	42.0%	1.14 (.62, 2.09)	1.74 (.98, 3.07)	1.08 (.58, 2.02)	1.90 (1.07, 3.38)*

* *p* < .05, **p < .01, and ***p < .001.

^a^Adjusted for age, education, and partnership status.

^b^Model did not converge (NC) due to zero observations in one or more conditions. *Note.* Odds ratios equal to 1.00 indicate the reference group. Each row represents a separate statistical model.

### Prevalence of Sexual, HIV, and Financial Care-seeking Behaviors

Table 4 describes the proportion of WESW engaging in various sexual risk, HIV care-seeking, and financial care-seeking behaviors. Sexual risk-taking was high, but HIV-care seeking behaviors varied among women. Most WESW reported having one or more acts of condomless sex in the past 3 months (79%, *n* = 429) and one or more acts of sex under the influence of drugs and/or alcohol in the past 3 months (73%, *n* = 394) (Table 4). The mean number of reported sex acts in the past 3 months without a condom and with drugs/alcohol was 9.4 (±16.9) and 5.7 (±7.9), respectively. Approximately one third of WESW were HIV-positive (35%, *n* = 192), of which 97% (*n* = 186) had initiated ART at the time of study enrollment. Among HIV-negative WESW, only 17% (*n* = 58) had initiated PrEP. Most HIV-negative WESW had taken an HIV test within the past 3 months (87%, *n* = 304). Financial care-seeking behaviors varied among women. Most WESW reported having asked for money from family members (88%, *n* = 478), but few had sought financial resources from formal lending institutions (13%, *n* = 71).

**Table 4.** Prevalence of Reported Sexual Risk, HIV Care-Seeking, and Financial Care-Seeking Behaviors Among Women Employed by Sex Work at Time of Study Enrollment (*N* = 542).

Sexual/HIV-Related Characteristics	*N*	(n/*N*) %
Total sample	542	100
Sexual risk behaviors		
≥1 act(s) of condomless sex^a^		
Yes	429	79.2
No	113	20.9
Mean number of condomless sex acts (±SD)^a^	9.4 (±16.9)	
≥1 acts of sex with alcohol/drugs^a^		
Yes	394	72.7
No	148	27.3
Mean number of sex acts with alcohol/drugs (±SD)^a^	5.7 (±7.9)	
HIV care-seeking behaviors		
Known HIV-positive status		
Yes	192	35.4
No	350	64.6
Among HIV-positive WESW, initiated ART		
Yes	186	96.9
No	6	3.1
Among HIV-negative WESW, initiated PrEP		
Yes	58	16.6
No	292	83.4
Among HIV-negative WESW, tested for HIV in last 90 days		
Yes	304	86.9
No	46	13.1
Financial care-seeking behaviors		
Asked for money from family members		
Yes	478	88.2
No	64	11.8
Asked for money from lending institution		
Yes	71	13.1
No	471	86.9

^a^Based on two most recent partners in last 90 days.

### Sexual, HIV, and Financial Care-seeking Behaviors Associated with Economic Abuse

Table 5 reports the crude and adjusted odds ratio (OR) of reported economic abuse by WESW’s sexual, HIV, and financial care-seeking behaviors. Given the high prevalence in any condomless sex (79%) and sex while high/drunk (73%), we did not observe a significant association between any condomless sex (OR = 1.43, 95%CI: .85–2.42) or any sex while high/drunk (OR = 1.41, 95%CI: .87–2.28) with reported economic abuse, respectively, in adjusted analyses (Table 5). However, the mean number of reported condomless sex acts in the past 3 months (*β* = +4.43, 95%CI: .44–8.41, *p *< .05) and the mean number of reported sex acts under the influence of drugs/alcohol in the past 3 months (*β* = +2.33, 95%CI: 0.71–3.94, *p* < .01) were both significantly higher among WESW experiencing high economic abuse compared to those experiencing low economic abuse. In contrast, despite higher sexual risk-taking, WESW experiencing high economic abuse had significantly lower odds of initiating PrEP (OR = .39, 95%CI: .17–.89) compared to WESW with lower economic abuse in adjusted analyses. WESW experiencing high economic abuse were also significantly more likely to ask family members for cash (OR = 2.36, 95%CI: 1.13–4.94) and seek financial resources from formal lending institutions (OR = 2.12, 95%C: 1.11–4.03) as compared to WESW with lower reports of economic abuse. ART initiation and HIV testing prevalence were relatively high (97% and 87%, respectively) with limited variability in the sample of WESW. No significant association was observed between economic abuse and initiation of ART (OR = .21, 95%CI: .02–2.22) or receipt of HIV testing (OR = 1.29, 95%CI: .58–2.90).

**Table 5.** Crude and Adjusted Odds Ratios (OR) of Sexual Behavior and HIV-Related factors Associated with Reported Economic Abuse (EA) in Women Employed by Sex Work by Total and by Level of Exposure (*N* = 542).

	By Any EA			By Level of EA						
	Yes	No	Crude OR (95% CI)	Low	Med	High	Crude OR (95% CI)		Adjusted OR (95% CI)^a^	
	%	%	Any versus None	%	%	%	Med Versus Low	High Versus Low	Med Versus Low	High Versus Low
Total (*N*)	503	39	—	189	178	175	—	—	—	—
Condomless sex^b^										
No	91.2%	8.8%	1.00	41.6%	31.0%	27.4%	1.00	1.00	1.00	1.00
Yes	93.2%	6.8%	1.35 (.63, 2.88)	33.1%	33.3%	33.6%	1.37 (.83, 2.26)	1.51 (.90, 2.53)	1.11 (.66, 188)	1.43 (.85, 2.42)
Sex while drunk^b^										
No	92.6%	7.4%	1.00	38.5%	31.8%	29.7%	1.00	1.00	1.00	1.00
Yes	92.9%	7.1%	1.06 (.51,2.21)	33.5%	33.3%	33.3%	1.21 (.76, 1.93)	1.31 (.82, 2.11)	1.37 (.84, 2.23)	1.41 (.87, 2.28)
Mean # condomless sex acts (±SE)	9.8 (±3.3)	4.3 (±3.2)	+5.55^c^ (−.91, 12.00)	7.0 (±1.4)	9.6 (±2.0)	11.6 (±2.0)	+2.53^c^ (−1.44, 6.50)	**+4.61**^c^ (.63, 8.59)*	+2.00^c^ (−2.04, 6.03)	**+4.43**^c^ (.44, 8.41)*
Mean # sex acts while drunk/high	5.8 (±1.3)	4.5 (±1.3)	+1.31^c^ (−1.28, 3.90)	4.9 (±.6)	5.3 (±.8)	7.0 (±.8)	+.38 (−1.24, 2.00)	**+2.09**^c^ (.46, 3.72)*	+.83^c^ (−.80,2.46)	**+2.33**^c^ (.71, 3.94)**
Initiated ART^d^										
No	83.3%	16.7%	1.00	16.7%	33.0%	50.0%	1.00	1.00	1.00	1.00
Yes	93.0%	7.0%	2.66 (.29, 24.50)	39.8%	29.6%	30.6%	.37 (.03, 4.20)	.26 (.03, 2.53)	.44 (.03, 5.78)	.21 (.02, 2.22)
Initiated PrEP^e^										
No	92.5%	7.5%	1.00	30.8%	33.9%	35.3%	1.00	1.00	1.00	1.00
Yes	94.8%	5.2%	1.52 (.43, 5.39)	41.4%	37.9%	20.7%	.92 (.45, 1.86)	.39 (.18, .89)*	.89 (.44, 1.82)	.39 (.17, .89)*
Received HIV test^f^										
No	93.5%	6.5%	1.00	39.1%	28.3%	32.6%	1.00	1.00	1.00	1.00
Yes	92.8%	7.2%	.94 (.26, 3.34)	31.6%	35.5%	32.9%	1.66 (.73, 3.75)	1.34 (.61, 2.93)	1.74 (.76, 4.01)	1.29 (.58, 2.90)
Asked relative for $										
No	92.1%	8.0%	1.00	37.0%	31.6%	31.4%	1.00	1.00	1.00	1.00
Yes	98.4%	1.6%	5.46 (.73, 40.7)	18.8%	42.2%	39.1%	2.61 (1.26,5.40)**	2.45 (1.18,5.10)*	2.42 (1.15, 5.08)*	2.36 (1.13, 4.94)*
Asked lender for $										
No	92.4%	7.6%	1.00	36.3%	33.3%	30.4%	1.00	1.00	1.00	1.00
Yes	95.8%	4.2%	1.88 (.55, 6.20)	25.4%	29.6%	45.1%	1.25 (.64, 2.47)	2.18 (1.16,4.11)*	1.23 (.61, 2.47)	2.12 (1.11, 4.03)*

*p < .05, **p < .01, and ***p < .001. Each row represents a separate statistical model.

^a^Adjusted for age, education, and partnership status.

^b^Based on two most recent sex partners in last 90 days.

^c^Difference in the mean number of sex acts with 95% CI. *Note.* Odds ratios equal to 1.00 indicate the reference group.

^d^Analytical sample only includes HIV-positive WESW (*N* = 192).

^e^Analytical sample only includes HIV-negative WESW (*N* = 350).

^f^Includes WESW who report missing >5% days with one or more missed medication doses in last 30 days.

## Discussion

This study examined prevalence of economic abuse among women employed by sex work in Uganda and associated demographic, sexual, and care-seeking characteristics with reported levels of economic abuse. Our study found economic abuse was a common experience of WESW. Prevalence was high (93%) with most WESW having experienced at least one form of lifetime economic abuse by an intimate partner, spouse, or family member. The three most frequently endorsed items (58%–80%) were abusers’ attempts to control women’s resources by requiring that they ask for money, keeping financial information from them, and excluding them from financial decisions. Most worrisome, a quarter to nearly half of WESW (24%–45%) reported experiencing employment sabotage with threats and demands to quit their job, physical violence when voicing a need to go to work, and/or financial exploitation from an abuser who created debt in their name or depleted existing resources. These findings are consistent with results reported in other studies that highlight the high prevalence of economic abuse and its detrimental effects on women’s social, health, and economic development (Davila et al., 2017; Tenkorang & Owusu, 2019; Stylianou et al., 2013; Yau et al., 2020). Strategies intending to reduce sexual and social risks of WESW should aim to improve WESW’s ability to prevent and safely respond to experiences of economic abuse.

In particular, our results highlight four important programming and research implications. First, we found that a significantly higher proportion of WESW reported economic abuse by their sex partners, including spouses, as compared to their family members for nearly all of the economic abuse items (11 out of 12). This may be attributed to sexual partners using financial resources to maintain ties within intimate relationships in absence of kinship ties. In fact, previous studies suggest that abusers acquire a sense of security from controlling their sexual partners and preventing them from leaving the relationship (Yau et al., 2020). In heterosexual couples, research has suggested that economic abuse in intimate relationships also occurs as a manifestation of men’s superiority and power over their female partners (Tenkorang & Owusu, 2019). This may explain why our study also found that married WESW and WESW with a sex worker boss (predominately male) were significantly more likely to report high economic abuse compared to single and self-managed WESW. It is possible also that married WESW had more shared responsibilities relating to household expenses that led to increased risk of abuse. What is evident from our findings is that economic abuse within and outside of sex work is a salient issue for many WESW. More efforts are needed to design and implement economic abuse prevention programs that target couples, sexual networks, and family units. Our findings suggest in particular that intimate partner-focused initiatives to raise awareness with male partners and sex work employers are of greatest need to reduce economic abuse by targeting abusers themselves.

Second, this study found that economic abuse was associated with other financial measures. WESW with below average income, those with debt, and those engaged in financial care-seeking (i.e., cash requests from family members or lending institutions) were more likely to report high economic abuse than higher-earning and debt-free WESW. Given the cross-sectional design of the study, it is unknown whether economic abuse is a consequence of lower income and financial care-seeking or whether lower income and financial care-seeking are consequences of economic abuse—or both. It is conceivable that higher-earning WESW can more confidently demand financial autonomy and may be more likely to live in households with more financial discretion to cover competing spending decisions—thereby minimizing tension and abuse. It is also plausible that WESW who are indebted to others are less able to prevent economic abuse by partners who view these behaviors as part of managing their debt. More research is needed to understand the directionality of economic abuse and other financial measures. Preliminarily, our results suggest that programs and policies that facilitate access to financial services in tandem with initiatives to minimize economic abuse, as a cause or consequence, are likely to be important strategies for female sex worker populations.

Third, high economic abuse among WESW was associated with significantly higher HIV vulnerability in the form of more frequent condomless sex and sex while high/drunk, as well as significantly lower uptake of PrEP in HIV-negative WESW. These are important behavioral measures as unprotected sex and the intoxicating effects of substance use prior to sex are established risk factors for unintended transmission of HIV (Calsyn et al., 2010; Hoffman, 2014). Consistent use of PrEP is also an effective biomedical intervention that can dramatically reduce HIV incidence (Baeten et al., 2012; Grant et al., 2010). This is a potentially worrisome finding although more research is needed to better understand the links between economic abuse and HIV risk, including directionality. Male condoms are relatively inexpensive in Uganda, and access to PrEP is free to eligible individuals in the country (Ministry of Health, 2016). However, our study found that WESW with higher economic abuse had lower monthly individual income which may exacerbate their ability to purchase condoms and/or pay for additional travel to PrEP-providing clinics. Other research has suggested that the resulting anxiety and stress resulting from economic abuse may also diminish women’s ability to negotiate condom use or to prioritize PrEP initiation (Tenkorang & Owusu, 2019). The high prevalence of economic abuse and high HIV infection in WESW in this study underscores the importance of integrating financial empowerment in HIV prevention interventions for WESW and their families, including education about the tactics of economic abuse and strategies to address them. Programs focusing on violence and abuse in women should also consider potential increased barriers to accessing and utilizing HIV prevention tools and services.

It is worth pointing out that in our analysis a binary measure of ever having condomless sex or sex while high/drunk or not in the last 3 months did not vary substantially in this sample and, therefore, did not function as a useful marker of sexual risk in this population. HIV testing in HIV-negative WESW and initiation of ART in HIV-positive WESW were also very high (87% and 97%, respectively) and did not vary in our sample. Low variability in these variables may have contributed to unobserved associations. If there is a true association between economic abuse and ART initiation and/or HIV testing, it may be more detectable within a larger sample or sample with a wider range of HIV testing and ART initiation behaviors. It is likely for this reason that when using a more variable, continuous measure of sexual risk-taking (i.e., frequency of acts) that the study was able to detect significant increases in the odds of high economic abuse.

Finally, we also observed differences in risk factors by level of reported economic abuse within the analysis’ high, medium, and low tertiles. Our results suggest preliminarily that the negative associations of economic abuse may be dose-responsive and/or cumulative. Reports of sexual risk-taking and financial care-seeking in WESW increased incrementally as the level of economic abuse increased, while HIV preventive care-seeking (i.e., PrEP) decreased incrementally as the level of economic abuse increased. More research in the form of larger and longer studies are needed to develop sensitive tools to measure incremental exposures to economic abuse. However, in resource-limited settings, it may be that WESW experiencing the highest levels of economic abuse are in greatest need of health, social, and financial support.

### Limitations

The limitations of this study should be noted. Given the cross-sectional nature of the study design, we were unable to make causal inferences regarding the directionality in the association of economic abuse and the range of demographic, sexual, and care-seeking measures. It is possible that there is a bidirectional, cyclical relationship between these measures as economic abuse was assessed in WESW’s most recent sex partners (or family members) and may have occurred prior to, during, or after the reported HIV-related behaviors. Therefore, it should be noted that all results are cross-sectional associations requiring more directional, longitudinal analysis. It is conceivable also that there are additional unmeasured variables that explain observed relationships. The analysis also did not qualitatively assess the context of economic abuse. Another limitation is that although WESW were encouraged to freely report experiences of economic abuse, it is possible that abuse was underreported due to lack of awareness if certain items were perceived as innocuous normal occurrences. Although the economic abuse items were drawn from previous research and showed high internal reliability in this study, additional assessment item construct validity in WESW would strengthen interpretations of future analyses. Lastly, WESW enrolled in the study represented individuals who were willing to participate in a randomized clinical trial and may therefore be less representative of more hard-to-reach WESW. Nonetheless, the study’s strengths include measuring a wide range of economic abuse items with high internal reliability by two types of abusers. The study is novel likewise in its assessment of economic abuse specifically in WESW and in the context of economic empowerment and HIV prevention in Uganda.

### Discussion of Diversity

The nature of this study addresses issues of diversity in several ways. One, the nature of the sample includes women employed by sex work from all aspects of socioeconomic status, sexual orientation, religion, age, partnership status, and geography in Uganda. Two, the measurement framework of the study assesses multiple perspectives and experiences of economic abuse among study participants, including representing measures across the current literature and practice of examining economic abuse. Three, as such, the study’s analysis expressly accounts for diversity in demographic characteristics (i.e., age, education, partnership status, etc.) and diversity of experience of economic abuse by type and level—in addition to including diverse HIV-related behavioral measures (i.e., condom use, initiation of antiretroviral medications). Lastly, the findings and observations of this study have the potential to be transferrable and generalizable to other diverse settings with women employed by sex work in sub-Saharan Africa.

## Conclusion

Our findings are consistent with results reported in other studies that highlight the high prevalence of economic abuse and its detrimental effects on women’s social, health, and economic development. More efforts are needed to design and implement economic abuse prevention programs that target couples, sexual networks, and family units. In addition, the high prevalence of economic abuse and high HIV infection in WESW in this study underscores the importance of integrating financial empowerment in HIV prevention interventions for WESW and their families, including education about the tactics of economic abuse and strategies to address them. Programs focusing on violence and abuse in women should also consider potential increased barriers to accessing and utilizing HIV prevention tools and services.
